# Positive Correlation Between Heavy Alcoholic Drinking and SARS-Cov-2 Non-Infection Rate

**DOI:** 10.7759/cureus.40130

**Published:** 2023-06-08

**Authors:** Ning-Hua Tong, Pietro Salvatori

**Affiliations:** 1 Department of Physics, Renmin University of China, Beijing, CHN; 2 Head and Neck Surgery, Consultancy, Milan, ITA

**Keywords:** public health, alcoholism, ethanol, disinfection, sars-cov-2

## Abstract

Introduction

During the SARS-CoV-2 pandemic, rumors claimed that alcohol drinking could someway be useful in contrasting the contagion and even the disease. It appears opportune to bring some robust data to determine whether heavy alcohol drinkers and non-drinkers experienced different infection rates.

Methods

A cross-sectional study through a simple survey based on the social media software Weixin and the mini survey program Wenjuanxing was carried out in China after the zero-Covid policy ended, namely from 15:00 January 1, 2023, to 12:35 January 3, 2023. The evaluation was conducted among subjects belonging to the first author's Weixin community, mostly residents in the higher populated China area. Study participants received a questionary and were asked about their virus infection status, and were classified into two groups: (a) infected, meaning he/she has been infected at least once (whether recovered or not); (b) remain uninfected, meaning the virus has not infected him/her. A total of 211 subjects adhered to the survey. Alcoholic drinking behavior about liquors with no less than 40% alcohol content in volume was retrieved from the participants. In China, such beverages are almost uniquely referred to as the Chinese Spirits or BaiJiu. The frequency of drinking quantified the drinking behavior, and it is classified into three groups: never drink or drink occasionally (group A); drink one or two times per week (group B); drink three times per week or more often (group C). The hypothesis of an existing relationship between infection status and drinking behavior was advanced before data collection. The numbers of the uninfected people in each of the three drinking groups were counted, and the rates of not-infection were calculated. The rates are compared with each other to conclude whether significant differences exist, considering the size of the samples. The conclusion is drawn from standard hypothesis testing.

Results

The male/female ratio was 108/103 (51.2% and 48.8%), the mean age was 38.8 years (range 21-68), and the median age of 37.4 years. The total 211 participants fell into three groups with different drinking frequencies, with counts (percentages in total 211 participants) 139 (65.9%) in group A, 28 (13.3%) in group B, and 44 (20.8%) in group C. The number (percentage within the group) of uninfected members in groups A, B, and C are 29 (20.9%), 7 (25.0%), and 17 (38.6%), respectively. The statistical analysis through the Cochran-Armitage trend test gave a significative result: p=0.0209.

Conclusions

Within the methodology's limitations, this study shows the significant relationship between alcohol drinking habits and the chances of avoiding SARS-CoV-2 infection. A possible hypothesis explaining these findings is advanced. However, the authors warn about misleading conclusions and advocate research that could properly guide ethanol use in the present and other possible pandemics.

Limitations

This study is based on self-reported data from a specific community in China. There could be recall bias and social desirability bias, and the generalizability of the ﬁndings to other populations could be limited. Other factors that could influence infection rates, such as age, occupation, and health status, are not controlled in the present study. There could be other explanations for the observed relationship between alcohol drinking habits and infection rates.

## Introduction

Coronavirus disease 2019 (COVID-19) has, up to now, led to hundreds of millions of infections and more than 6.5 million deaths worldwide [1]. Numerous studies have been devoted to methods that could effectively prevent infection of SARS-Cov-2. Besides the debatable role of vaccination, certain practices in everyday life, such as wearing a mask, social distancing, and washing hands, are believed to play positive roles in preventing infection [2]. For example, wearing a mask has been confirmed to be able to reduce the risk of virus transmission [3]. Ethyl alcohol, or ethanol (EtOH), has been confirmed to strongly affect the virus outside the human body [4, 5]. There are some statements that alcohol intake has no positive effect on preventing the infection of the virus that causes COVID-19 [6]. The negative impact of alcohol drinking on health in the time of the COVID-19 pandemic has been discussed extensively [7]. However, an opposite report found that US counties with high alcohol consumption and high rurality experienced a significantly lower COVID-related mortality rate [8].

In summary, except for the well-known negative effect of alcohol on human health in general, to our knowledge, the correlation between liquor drinking and the rate of infection (or not-infection) of SARS-Cov-2 has not been studied seriously. On the other hand, some reports elucidated the theoretical bases of the EtOH efficacy in eradicating the SARS-Cov-2 from airways [9], the efficacy in preventing infection [10], and improving COVID-19 outcomes [11, 12]. With the end of the dynamic zero-COVID policy in China on Dec 7, 2022, a tide of infection emerged, and the number of people infected with SARS-Cov-2 increased rapidly in China in January 2023. Most of the infections are proven by antigenic self-test at home. The situation of a large number of infections and the availability of self-test kits for the public provides a unique opportunity to study the correlation between the infection rate of SARS-Cov-2 and certain interesting behaviors of everyday life in the population.

In this paper, we report the investigation on the correlation between the infection (or not-infection) rate of SARS-Cov-2 and heavy alcoholic drinking, carried out in a specific period after the end of the zero-COVID policy and in the restricted population of China. The purpose of the present paper is to report the investigation of the two incidents, i.e., liquor drinking and virus infection, and to discuss the eventual correlations. Various possible explanations for the observed correlation are provided and analyzed. 

## Materials and methods

A cross-sectional study aimed to investigate the correlation between heavy alcohol drinking and SARS-Cov-2 (referred to as the virus below) infection was carried out in China using a questionary survey. The questionary was generated by the mini program Wenjuanxing, which is included in the social media platform Weixin (the Chinese version of WeChat), and was circulated among the first author's contacts, distributed in various Weixin groups, mostly residents in the higher populated China area. Circulation started at 15:00, Jan 1, 2023, and data were collected at 12:35, Jan 3, 2023. Wenjuanxing is a free, easy-to-use, and anonymous survey Weixin mini program widely used in China. 

The detailed procedure of the survey is as follows: (1) We designed the questionnaire and made an online sheet using the mini-software Wenjuanxing, a commonly used survey tool in China; (2) We released the link of the questionnaire to some Weixin groups to which the first author belongs; (3) We explained the purpose of this survey, promise anonymity, and promise that the result of the investigation, once obtained, will be released to the group; (4) We called for participation and encouraged the members of the group to share and spread the link; (5) We monitored the number of data collected and how it changed with time; (6) Once the number of respondents did not change much over one day, we stopped collecting data and download the data from Wenjuanxing.

The questionnaire was designed to be as simple as possible, concerning only two facts of the investigated individuals, namely: the status of virus infection and the behavior of liquor drinking. All participants were asked to assess their infection status by self-administered antigenic rapid test and choose one of the answers to each of the following two questions: 1) "What is your present infection status? (a) infected (recovered or not); (b) remain not-infected", and 2) "What is your frequency of drinking strong liquor (containing no less than 40% alcohol in volume)? (a) never or occasional; (b) one or two times per week; (c) three times per week or more often".

According to the answer to the first question, participants were classified into two groups: (a) infected, meaning they had been infected at least once (whether recovered or not); (b) remain not-infected, meaning they had not been infected by the virus. Note that due to the dynamic zero-COVID policy carried out in China before Dec 7, 2022, the number of those infected before that date or who have been infected more than once is negligibly small. In this questionnaire, we, in practice, did not specify and discern the time range of infection and the number of infections. Several issues regarding the data of this variable, including the accuracy of self-administered antigenic rapid test, possible recall bias, and social desirability bias, and their possible influences on the conclusion of the present study, are analyzed in the discussion part.

As for the alcoholic drinking behavior, we only focused on strong liquor, which is defined as alcoholic beverages with no less than 40% alcohol content in volume. In China, such beverages are almost uniquely referred to as Chinese Spirits or Baijiu. The spirit is usually drunk at room temperature but is also acceptable if warm. Drinking behavior is quantified by the frequency of drinking, and it is classified into three groups: (group A) never drink or drink occasionally, (group B) drink one or two times per week, and (group C) drink three times per week or more often. For simplicity, other aspects of drinking behavior, such as the amount of drinking and the way of drinking (alone, with family, or with friends), are not investigated by the questionnaire. However, according to the personal experience of the first author, a typical scenario of regular drinking in group C is drinking in moderation (about 50-150 grams of liquor), at lunch or dinner time, with family or close friends.

Note that the drinking behavior is quantified by the frequency of drinking instead of the amount of EtOH since it is believed that, if drinking alcohol is indeed associated with the change of risk in virus infection, it must be the frequency instead of the total amount that is the decisive factor. Also, under the assumption that the content of EtOH could influence the risk of infection - the higher the content of EtOH, the lower the risk of infection - we only investigated strong liquors. Due to the difficulty of accurately estimating the frequency of drinking for many individuals, a more detailed classification of frequency was judged not reliable. To simplify the study, other variables that could be associated with the infection rates, such as age, occupation, and health status, are not controlled. The possible influence of these factors on the result is discussed in the discussion part.

It is hard to estimate the number of questionnaires distributed since the questionnaire could have been transferred from one Weixin group to another or from person to person. The response rate, calculated by dividing the number of responses by the estimated total number of people in the Weixin groups where this survey link was released, is about 10%. Finally, a total of 211 questionnaires met the inclusion criteria. These participants are believed to be those who are interested in this investigation and are motivated by pure curiosity about the result. All participants gave verbal consent, and basic demographics were noted. 

The association between infection status (infected or not-infected) and the three drinking groups was statistically investigated through the Cochran-Armitage trend test. Furthermore, for descriptive purposes, we computed ratios of non-infection rates for "one to two times per week" (group B) and "three or more times per week" (group C) drinking frequency categories relative to the "never, occasionally" (group A) category and corresponding 95% asymptotic confidence limits.

## Results

**Figure 1:**
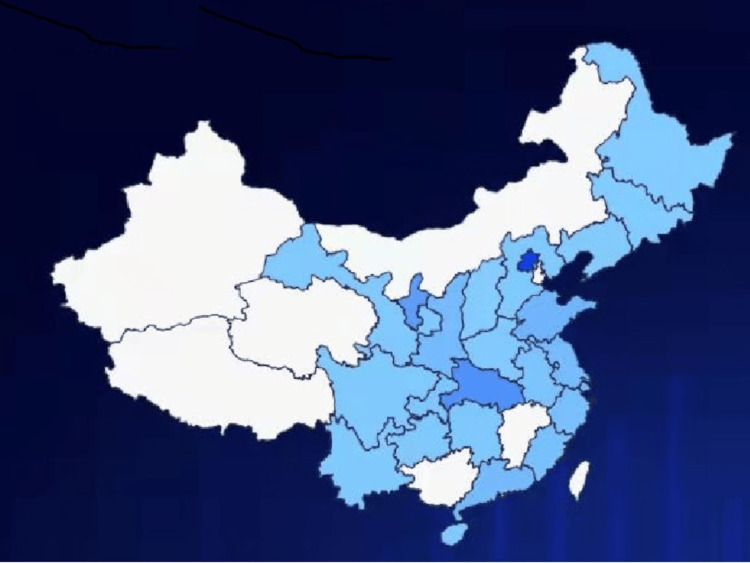
Geographic distribution of the collected questionnaires in China. Darker blue corresponds to a larger number

Figure 1 shows the geographic distribution of the 211 respondents in China. Although the spread and distribution of the questionnaire are hard to control and are influenced by the social community of the first author, it is seen from Figure 1 that the data are collected from the most heavily populated area of China. The darkest area on the map is Beijing, the city where the first author works and lives. The male/female ratio was 108/103 (51.2% and 48.8%), the mean age was 38.8 years (range 21-68), and the median age was 37.4 years.

**Figure 2:**
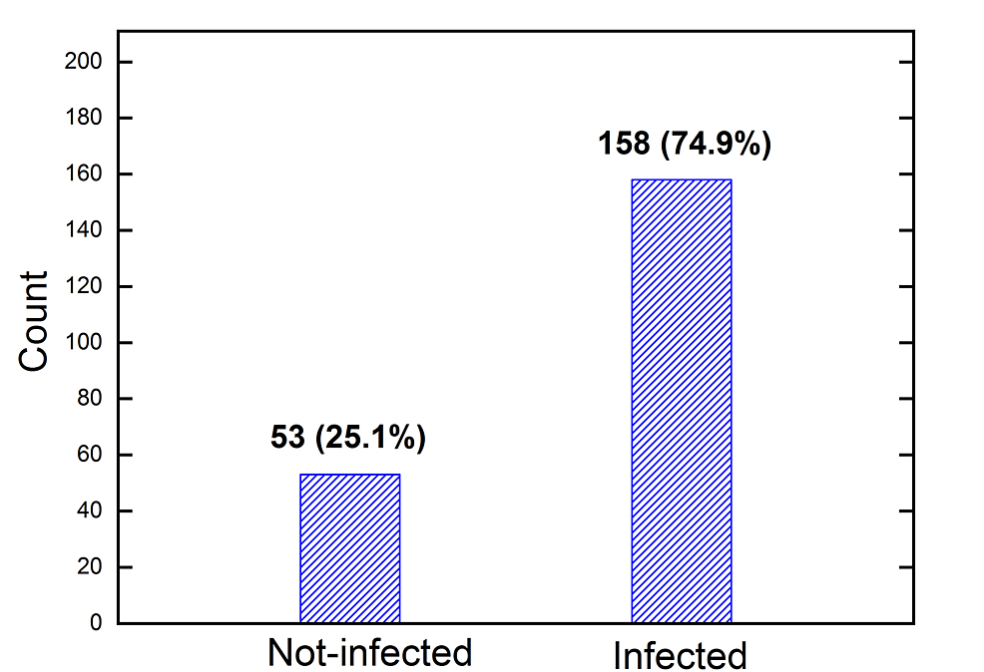
Count of not-infected people and infected people in the total 211 collected questionnaires. The percentages of the total 211 are shown in the brackets

Figure 2 shows the distribution of the total 211 respondents according to the infection status: 53 (25.1%) were not-infected, and 158 (74.9%) were infected. There was no official data for the infection rate at the time of the study. Our result of 74.9% is consistent with estimations from various sources that range from 60% to 80%.

**Figure 3:**
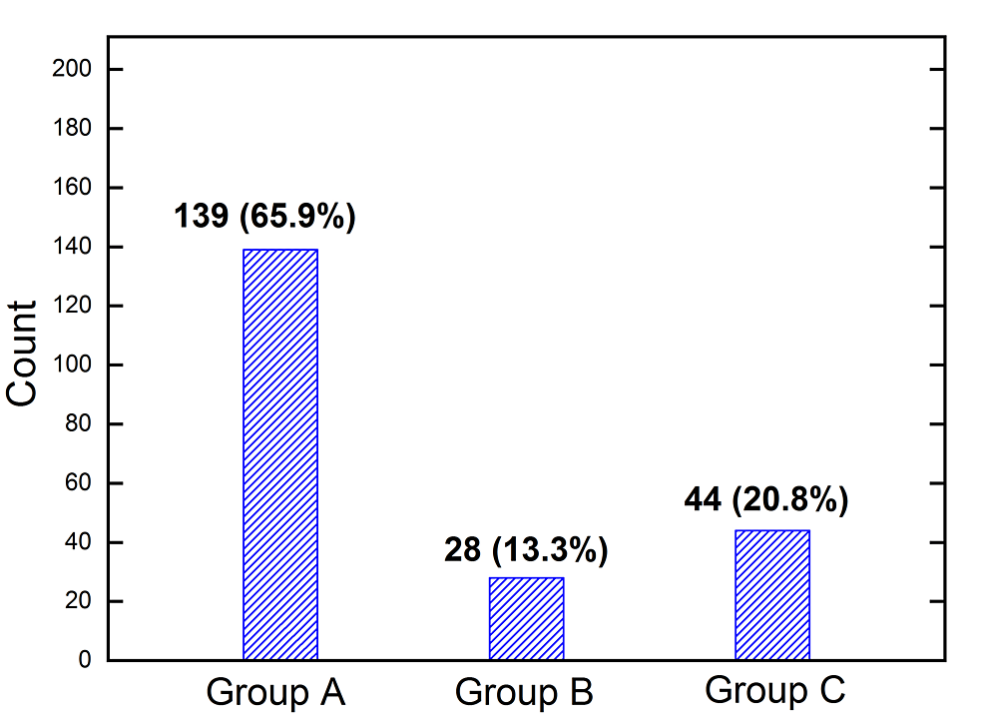
Count of the number of respondents in the three groups with different liquor drinking-frequencies. The percentages in the total 211 are shown in the brackets

Figure 3 displays the distribution of the total 211 participants according to drinking behavior: 139 (65.9 %) in group A, 28 (13.3 %) in group B, and 44 (20.8 %) in group C. 

Table 1 summarizes the number of infected and not-infected respondents in each of the three groups with different drinking frequencies. Albeit the number of not-infected respondents varies in each group, the percentage of it in each group shows an interesting trend: it increases from 20.9% in the nondrinker group (A), to 25.0% in the mild drinker group (B), and a much higher value of 38.6% in the heavy drinker group (C). The average not-infection rate of the total 211 respondents - 25.1% - is close to the value of the mild drinker group (B).

**Table 1:** The count of infected and not-infected respondents in the three drinking groups

	Never or occasionally (A)	1-2 times per week (B)	≥ 3 times per week (C)	Total
Not-infected	29 (20.9%)	7 (25.0%)	17 (38.6%)	53 (25.1%)
Infected	110 (79.1%)	21 (75.0%)	27 (61.4%)	158 (74.9%)
Total	139	28	44	211

**Figure 4:**
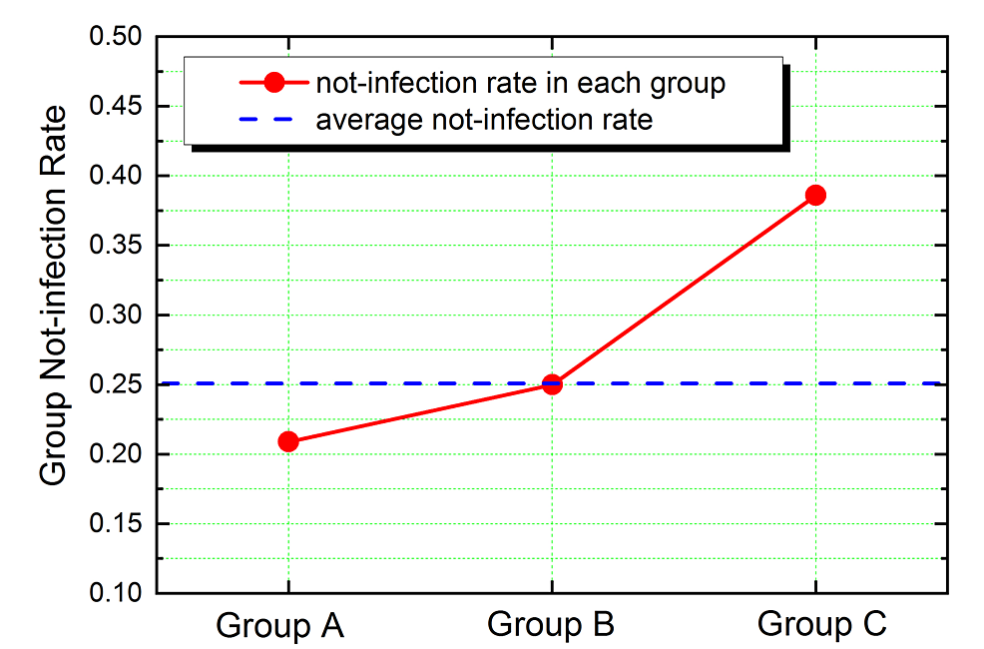
The not-infection rate as a function of liquor drinking frequency (red dots with eye-guiding line). The blue dashed line shows the total not-infection rate from the whole sample of 211 respondents.

The trend of the data in Table 1 is shown in Figure 4 as a monotone-increasing curve.

This is a simple result from the survey: the more frequently people drink liquor, the lower risk they have of being infected with the virus. It is remarkable that the not-infection rate of 38.6% of the heavy drinker group almost doubles the value of 20.9% of the nondrinker group. The two-sided Cochran-Armitage trend test gave a significative result: p=0.0209. Non-infection rate ratios with 95% confidence limits were group B) vs. group A, 1.1983 (0.5684-2.3099); and group C vs. group A, 1.8519 (1.1123-2.9781).

## Discussion

Overall, Figure 1 shows that the collected data are representative of the situation of the whole country.

We have observed in Figure 4 an apparent correlation between heavy alcohol drinking and a higher not-infection rate. Below, we discuss several issues related to the quality of our data and critically examine whether these issues can influence the validity of the observed correlation. Once we can establish the correlation on a solid basis, we then discuss various possible explanations for it. Among all the possible explanations, we propose the most probable one based on the present knowledge.

The following issues are related to the quality of our data

Size of the Sample

The size of our sample of 211 in this study could raise concerns about the statistical significance of the observation. In a statistical study, whether a sample size is sufficient or not is determined by its ability to discern the signal from accidental fluctuations and is commonly judged by the p-value. In our case, the contrast between the not-infection rates of the non-drinker group and the heavy-drinker group is relatively large, so the sample size of 211 is already sufficient to identify the contrast and to rule out the possibility of it being due to accidental fluctuations, as shown by the small p-value of 0.0209.

Willingness to Respond in Different Groups

Since the response to the questionnaire is volunteered, in principle, there could be a deviation in the response willingnesses of the infected and uninfected people, and this deviation could differ between the heavy-drinker group and the non-drinker group. If this is the case, the data for the infected and not-infected rates obtained in each group will be inaccurate, and it will influence the validity of the correlation. To see to what extent this possible situation will influence our conclusion, we make the following analysis on the practical inference principle. Let us assume that there is no correlation at all between the not-infection/infection rate and the drinking behavior and that the observed correlation in Figure 4 is purely due to the above-mentioned difference in the deviations of response willingness. The simple analysis then indicates that to obtain the data shown in Figure 4, in group A (non-drinker group), an infected member will have to respond to our questionnaire with a probability 1.26 times that of a not-infected member, while in group C (heavy-drinker group), an infected member will have to respond with about half the probability of that of a not-infected member. We find this highly improbable, given the fact that the present survey is anonymous and voluntary and that the participation is mostly motivated by pure curiosity about the investigation result (which we promised to announce), which is neutral on infection status and drinking behavior. We, therefore, conclude that this issue is unlikely the cause of the correlation observed in our data. 

Pollution of Samples 

Using Wenjunaxing's IP address data, we can largely exclude the possibility of repeated responses. The probability of a dishonest response is low because the response is volunteered. Most antigen rapid self-test in China has a true positive rate (TPR) of 75%-98% and a true negative rate (TNR) of 95%-99%. The inaccuracy in the reported infection/not-infection data due to this issue should not generate serious problems since, to a large extent, the effect of false positive cases is balanced out by false negative cases when both numbers are large. Moreover, the influence applies equally to the three groups of drinking behavior and has thus limited influence on the contrast between them, upon which our conclusion is drawn.

Bias of Samples

The investigation is limited to active users of the social media platform Weixin, effectively expelling younger, elder, seriously sick, and deceased individuals. However, unless confirmed data shows that these individuals have significantly different infection rates from the participants of the present survey, this issue does not seem to have much influence on our conclusion. We also expect that most of the heavy-drinking participants are in a perfectly sober state when doing the survey, considering that they need to handle the mobile phone and they do so purely out of curiosity about the investigation result. The issue of possible social pressure making heavy drinkers reluctant to reveal their infection status can be reduced to issue two).

Correlations in the Samples

The infection of family members and friends could be correlated. This, if true, will reduce the effective size of the sample and make our p-value underestimated. Considering that Figure 1 shows a wide geographical distribution of response and that IP addresses of response show no signature of clustering, we believe that this issue will not have a significant influence on the data. 

In summary, our analysis above shows that none of the above issues can severely damage the confidence of our conclusion, i.e., there exists a positive correlation between heavy alcohol drinking and a higher not-infection rate. It increases our confidence that the observed correlation is not an artifact created by bad data.

Possible explanations of the correlation

There could be many different ways to interpret the observed trend, and all of them are open for discussion. At the present stage, the data obtained from our study are still not sufficient to pinpoint any of them. However, based on probability, our data do assign different weights to the credibility of each interpretation. The final solution to this problem awaits the study on a larger scale and with better control of various factors. Possible interpretations include:

Heavy Drinkers Are Probably in a Drunken State Most of the Time. Therefore They Have Reduced Social Activity and Thus Effectively Reduce the Infection Rate

As we have analyzed above for issue four about the bias of the sample, there is no evidence showing that the heavy drinkers are in a drunken state most of the time, and thus, they have reduced social activity since the heavy drinkers in our study are defined by drinking frequency instead of by drinking amount. The fact that they interact on social media Weixin group and actively participate in the investigation supports that they are more likely to be drinkers in moderation. This interpretation, therefore, has lower credibility.

Heavy Drinkers Tend to Be More Solitary Drinkers

Our social experiences hint that many regular drinkers are not solitary drinkers. Instead, they developed a drinking habit at lunch or dinner time with their family or friend. So the loneliness of heavy drinkers cannot provide a satisfactory explanation for the trend that we observed, either.

Indirect Causal Relation Through Other Factors Such As Age, Occupation, Health Status, etc.

Certain groups of the population, such as people with distinct ages, occupations, or health statuses, could have more heavy drinkers and lower infection rates simultaneously for some reason. This could lead to the correlation observed in Figure 4. Our data, due to its simplicity, can neither support nor refute this interesting scenario. However, to our knowledge, up to now, there is very limited research data showing significant deviations in the infection rates of different groups of the population mentioned above. This interpretation still awaits further data support.

Direct Causal Relation Between the Ethanol Intaking and Higher Not-Infection Rate

This explanation fits in the current knowledge about the effect of ethanol on enveloped viruses such as SARS-CoV-2 and has some support from other experimental results. For details, see below. Our opinion is that, although our present data cannot unambiguously confirm this explanation, it is of higher level credibility among all the explanations and thus deserves serious consideration.

Others

Undoubtedly, we have not exhausted all possible explanations.

Before we present a possible mechanism for the causal relationship between heavy liquor drinking and the higher not-infection rate, it appears opportune to dwell on EtOH in the treatment and prevention of SARS-CoV-2 infection and COVID-19 in general, in light of recent studies in this field.

First of all, EtOH is a regular drug listed in the USA and EC pharmacopoeias and is mainly used for methanol and ethylene glycol poisoning. It has to be remembered that since the 1950s, inhalation of EtOH has been proven to be both safe and effective for treating coughs and pulmonary edema [13, 14]. Moreover, ethanol (up to 9 mg) is frequently used as an excipient in inhalation treatment for asthma and chronic obstructive pulmonary disease [15]. EtOH is also widely used in disinfection procedures. Its antiviral properties derive from the solvent effects on lipids (pericapsid, or envelope) and from the denaturation of proteins (capsid) [16]. Human coronaviruses, including severe acute respiratory syndrome coronavirus (SARS), Middle East respiratory syndrome (MERS), human endemic coronavirus, and influenza-A viruses, have been demonstrated to be significantly affected by ethanol on surfaces like plastic and glass, where these viruses can survive for days. Current experimental data show that an ethanol concentration of 30% v/v can inactivate SARS-CoV-2 in 30 seconds [17]. SARS-CoV-2 is an enveloped virus that is extremely sensitive to ethanol, which is also effective against all SARS-CoV-2 variants and other "enveloped" viruses due to its non-specificity. This particular characteristic broadens the ethanol's range of activity against the SARS-CoV-2 pandemic and suggests its use in potential future epidemics from "enveloped" viruses.

The quantity of EtOH required to reduce the SARS-CoV-2 viral load affecting the lungs was determined by Manning et al. [18], and it amounts to 153 μg or 191.25 μL. Elimination of EtOH occurs at a rate of 120 to 300 mg/L/hour [19]. Alcohol dehydrogenase breaks down 95% of EtOH that has been consumed (or breathed), while the remaining 5% is removed - unaltered - by exhaled air, urine, perspiration, saliva, and tears. Due to the large area of the alveolar-capillary interface, it seems reasonable to assume that one-fifth of the unaltered, active EtOH escaping the metabolic degradation is eliminated through this pathway. Then, in a normal adult, the amount eliminated through the air is 1% of 120-300 mg/l/hour, so 1.2-3 mg/L over one hour. Considering a normal respiratory frequency of 15 acts/min = 900 acts/hour, this means that each exhalation contains 0.0013-0.0033 mg = 1.3-3.3 μg of EtOH. Thus, the calculated dose of 153 μg for inactivating the viral load is approximately exhaled within 118-46 minutes.

Based on the above studies, a possible explanation of our results is that EtOH could reduce the odds of developing infection through a two-way pattern: 1) The EtOH that evaporates while drinking the spirit is inhaled and directly inactivates or destroys the virus lying over the naso-oro-pharyngeal mucosa, which is the most important point of entry for the virus [20], whereas the ingested ethanol that is eliminated through the lungs reaches the upper respiratory tract travelling within the exhaled air. The amount of the exhaled EtOH is probably lower than the inhaled one, but the continuity of respiration grants a longer action over time; and 2) The frequent washing of the throat by high concentration alcohol inactivates the virus by direct contact.

It is reasonable to suppose that all three events (inhaled, exhaled, direct contact) concur with the final finding in a synergistic way. Our findings match those showing that US counties with high alcohol consumption and high rurality experienced a significantly lower COVID-related mortality rate [8]. Interestingly, the significance of the Cochran-Armitage trend test supports our hypothesis that frequency matters more than the absolute quantity of ingested EtOH. Intuitively, recurrent actuation of the disinfectant improves the chances of inactivating a pathogenic agent. 

In summary for this part, among various possible explanations, we find it more probable and natural to attribute the observed correlation to the inactivation effect of EtOH on COVID-19. This explanation still requires verification or falsification by a further study on a larger scale.

Below, we discuss the prospect of EtOH in the treatment and prevention of COVID-19 in general, in the light of present findings and the literature in this field.

EtOH toxicity is usually - and in some way, improperly - considered an insurmountable obstacle for wider medical usage. First of all, there is a significant difference between ingested and inhaled ethanol since the latter bypasses the first necessary metabolic step of ingested ethanol and instead travels straight to the left ventricle of the heart and the brain [21]. Second, chronic ethanol use is not the same as chronic ethanol abuse, which can result in lung damage (alveolar macrophage dysfunction, and increased susceptibility to bacterial pneumonia and tuberculosis) [22]. Finally, chronic intoxication has to be differentiated from acute one.

On the topic of acute EtOH inhalation, Bessonneau [23] has demonstrated that the cumulative dose of ethanol inhaled in 90 seconds, while surgically disinfecting hands with a gel containing ethanol at a concentration of 700 g/l, is 328.9 mg. 

Rules governing acute ethanol exposure vary by nation or state and are subject to laws. The maximum Blood Alcohol Concentration (BAC) usually ranges between 500 and 800 mg/L.

The regulation also restricts the maximum amount of chronic ethanol exposure in the workplace. For instance, the occupational exposure limit (OEL) for ethanol in the United Kingdom is 1000 parts per million (ppm) of ethanol, or 1910 mg/m3, during an eight-hour shift, which is equivalent to consuming 10 g of ethanol (about one glass of alcohol) daily, according to estimates [24]. These numbers are in perfect agreement with Bessonneau's report [23], and much exceed the amount that would theoretically be needed to reduce the virus load in the respiratory tract [18]. The worries about EtOH inhalation appear to have been completely dispelled by the thorough study of Castro-Balado et al. [25]. Indeed, in rodents breathing, 65% v/v ethanol for 15 min every eight hours (three times a day), for five consecutive days (flow rate: 2 L/minute), these authors examined the possible mucosal or structural damages to EtOH in the lung, trachea, and oesophagus. In this experiment, the calculated absorbed dosage was 1.2 g/kg/day. Under the same conditions, this dosage in humans would be equivalent to 151 g per day. Notably, neither the treated animals nor the controls' histology samples showed any signs of damage. A recent randomized controlled trial (RCT) from the same group has confirmed these data in humans [12].

Interestingly, in the RCT from Hosseinzadeh [10], no collateral effects have been mentioned, perhaps because they were lacking or minimal and tolerable, whereas Amoushahi et al. reported few and bearable adverse events [11]. Finally, numerous studies suggest that industrial exposure is not a problem in reproductive medicine (Irvine) [26], nor in oncology (Bevan) [24], despite the toxicity of chronic ethanol inhalation.

In our opinion, papers intended to reasonably combat the inappropriate EtOH use in COVID-19 seem not to have taken due account the knowledge of EtOH, including toxicity. Indeed, the document released by WHO [6] is merely divulgation and does not bring any evidence supporting the section "general myths about alcohol and COVID-19" but simply affirms that "there is no evidence that drinking alcohol offers any protection against COVID-19 or has a positive effect on the course and outcomes of any infectious disease". Now, if this might have been acceptable in 2020, at present, the evidence showed by Pro et al. [8] should lead to a selective update, at least. Particularly, the RCT from the Spanish group [12] found a faster and higher - even not significative - reduction of the viral load in the EtOH-treated patient's group versus controls. Unfortunately, the RCT had to be ended earlier for insufficient recruitment.

It should also be remembered that aerosol delivery is more efficient than simple inhalation. Moreover, in the editorial from "Alcohol and Alcoholism" [7], the author reports a considerable number of deaths from "alcohol" intake during the pandemic. But, when examining the cited references, it appears clear that they refer either to methanol or other drugs added to "alcoholic beverages". Moreover, these papers report events that occurred in 2018 and 2019, then before the SARS-CoV-2 pandemic.

Due to these someway misleading messages, the medical body and health authorities could have neglected an efficacious, easily available treatment to help pandemic control.

Shintake [27] on March 17, 2020, and Amoushahi et al. [28] on May 25, 2020, are credited for first hypothesizing EtOH treatment to prevent or eradicate SARS-Cov-2 infection. Today, it seems there is sufficient research body leading to deeply test the EtOH efficacy and efficiency on airways disinfection in SARS-CoV-2-positive subjects and COVID-19 patients [9-12]. The positive correlation between heavy alcohol drinking and the SARS-Cov-2 not-infection rate reported in the present paper lends itself to the importance of such research.

Very interestingly, the recent work from Shintake's group [29] undoubtedly demonstrates that brief EtOH vapor inhalation twice a day protects mice from lethal influenza-A virus respiratory infection by reducing the viral load in the lungs without harmful side effects. According to the theoretical bases, these data suggest that EtOH vapor inhalation may provide a versatile therapy against various respiratory viral infectious diseases. 

It has to be made clear that the authors are deeply aware of the harmful potential of the EtOH and do not certainly support its oral intake for the prevention or treatment of SARS-CoV-2 infection and COVID-19 disease. However, they believe that a more reliable word on this topic is necessary for the correct public health management, either to offer an efficacious/efficient treatment or to avoid possible damages from the EtOH myth or misuse. Because science does not fight myth by replacing it with another myth.

## Conclusions

During the SARS-CoV-2 pandemic, rumors claimed that alcohol drinking could someway be useful in contrasting the contagion and even the disease. For us, it appeared opportune to bring some robust data to determine whether heavy alcohol drinkers and non-drinkers experienced different infection rates. The study we carried out is agile but implies some methodology limitations. Nevertheless, it shows the significant relationship between alcohol drinking habits and chances of avoiding SARS-CoV-2 infection. However, the authors strongly warn about misleading conclusions and advocate research that could properly guide ethanol use in the present and other possible pandemics. Ethanol is a powerful disinfectant, largely available and very cost-effective.
